# Development of an Information Guideline for Kidney Transplant Recipients in a Clinical Trial: Protocol for a Modified Delphi Method

**DOI:** 10.2196/46961

**Published:** 2023-11-06

**Authors:** Araceli Faraldo-Cabana, Ana Sánchez-Fructuoso, Isabel Pérez-Flores, Juan Vicente Beneit-Montesinos, Daniel Muñoz-Jiménez, Belén Peix Jiménez, Sara Asensio Arredondo, Enriqueta Isabel Nuño Santana, María José Santana Valeros, Virginia Hidalgo González, Fernando González García, Ismael Ortuño-Soriano

**Affiliations:** 1 Hospital Clínico San Carlos Instituto de Investigación Sanitaria San Carlos Madrid Spain; 2 Hospital Clínico San Carlos, Facultad de Medicina Universidad Complutense de Madrid Instituto de Investigación Sanitaria San Carlos Madrid Spain; 3 Facultad de Enfermería, Fisioterapia y Podología Universidad Complutense de Madrid Instituto de Investigación Sanitaria San Carlos Madrid Spain; 4 Hospital Clínico San Carlos Instituto de Investigación Sanitaria San Carlos Red de Investigación en Cronicidad, Atención Primaria y Prevención y Promoción de la Salud. Unidad de Investigación en Cuidados y Servicios de Salud del Instituto de Salud Carlos III Madrid Spain; 5 Hospital Clínico San Carlos Madrid Spain; 6 Hospital Ramón y Cajal Madrid Spain; 7 Hospital Doce de Octubre Madrid Spain; 8 Hospital La Paz Madrid Spain; 9 Hospital Gregorio Marañón Madrid Spain

**Keywords:** compliance, Delphi method, guideline, kidney transplantation, patient adherence, patients

## Abstract

**Background:**

Renal transplantation is the treatment of choice for most cases of end-stage renal disease. Recipients need to lead a healthy lifestyle to minimize the potential side effects of immunosuppressive drugs and improve transplant outcomes. There is not much evidence about the best way to increase adherence to healthy lifestyles in kidney transplant recipients, so one of the objectives set by the nursing team is to train people to acquire the necessary skills and tools to be able to take care of themselves. In this sense, the consensual development of appropriate materials may be useful and of interest.

**Objective:**

The aim of this study was to develop an information guide for adults with kidney transplants to be assessed in a subsequent clinical trial as an intervention to increase adherence to healthy habits.

**Methods:**

We used a 3-step, methodological, sequential approach: (1) training from a group of experts and item consensus; (2) review of the medical literature available; and (3) use of the Delphi technique with on-site meetings. A total of 5 nurses from the Community of Madrid Kidney Transplantation Unit in Spain were asked to participate. The patients’ lifestyle factors that, according to the medical literature available and experts’ opinions, have the greatest impact on the survival of the transplanted organ and the recipients themselves were all described.

**Results:**

After using the modified Delphi method to reach a consensus on the items to be included and the information needed in each, an information guide for adult kidney transplant patients was developed. This guide facilitates the structuring of health care, information, and recommendations necessary for effective self-care for each person. The result is considered to be an easy-to-understand tool, useful for transplant doctors and nurses, in simple language, with information based on the latest scientific-medical evidence published to date, aspects of which will be evaluated in a clinical trial designed for this purpose.

**Conclusions:**

Currently, this guide is the main intervention variable of a clinical trial (registered on ClinicalTrials.gov; NCT05715580) aimed at improving compliance with healthy habits in kidney transplant recipients in the Community of Madrid, Spain. The method used in its development has been useful and agile, and the result is a guide that can be easily updated periodically following the same procedure.

**International Registered Report Identifier (IRRID):**

DERR1-10.2196/46961

## Introduction

### Overview

Kidney transplantation is the treatment of choice for most cases of end-stage renal disease because this therapy brings better quality of life and prolonged survival. Also, in the long run, it is less expensive compared to dialysis [1-3].

In Spain, the number of kidney transplants has gone up over the past few years, from 52 transplants per million inhabitants back in 2011 to 62.3 transplants per million inhabitants in 2021 [4].

### Importance of Adherence to Healthy Habits and Medication

When someone undergoes a kidney transplantation, they need to change a few habits [5]. This, added to compliance with the drug therapy prescribed, is the key to a successful transplantation procedure [6,7]. Also, to prevent possible side effects associated with immunosuppressant medication and improve the results of transplantation, the patient needs to develop healthy habits [8-14].

According to the kidney transplantation protocol, health care providers should have the necessary tools to provide scientific evidence–based, updated, and quality information to implement measures to help patients with kidney transplantation improve their health-promoting lifestyles after the transplant [15]. This should result in longer survival rates for the organ, fewer complications, and lower health costs.

Promoting healthy habits should be encouraged by health professionals involved in patient care on a routine, daily basis since they can have an impact on the patients’ decision-making process in this setting [16,17].

### Relevance of Evidence and Consensus in Clinical Practice

The aim of developing this information guide for adult kidney transplant recipients is to increase compliance with healthy habits by harmonizing best practices, which are associated with better outcomes for both the organ and the recipient [18]. This guideline will provide the necessary information regarding self-care. Therefore, patients will be able to manage their own health-disease process and reduce the level of concern as they acquire knowledge on the entire transplantation process.

Also, this information guideline for adults with kidney transplantation will unify criteria regarding the scientific evidence–based information that should be given. Therefore, nurses from the kidney transplantation unit will be able to use this guideline to manage the information that should eventually be given to adult patients with kidney transplants by standardizing practice by espousing structured care and doing so in plain language.

### Contribution

The aim of this study was to develop a consensus document providing information for adults with kidney transplants, and this paper aims to present the elaboration process, which we believe has been appropriate and yielded robust results. The secondary objectives were (1) to describe the habits that have an impact on the proper functioning of the transplanted kidney and, therefore, on the survival of the latter, and (2) to identify the most relevant evidence regarding self-care for patients with kidney transplantation. The effect of the developed guideline is being evaluated in a large clinical trial (registered on ClinicalTrials.gov; NCT05715580) titled “Impact of Nursing Intervention on Adherence in Kidney Transplant Recipients.”

## Methods

### Overview

We used a 3-step sequential methodologic approach, as explained in this paper. It is worth mentioning that the development of this guide is part of a much broader research project whose objective is to evaluate the educational process using the information guide as the main tool. This is a clinical trial intended to increase the compliance of the adult population with kidney transplantation with drugs and healthy habits through an informative intervention implemented by the nursing team from the Community of Madrid Kidney Transplantation Unit in Spain. It is expected that by increasing compliance, clinical parameters indicative of poor renal function will improve as well.

### First Step: Training From a Group of Experts—Consensus and Item Decision-Making

In the first place, to develop the information provided by the guideline, a group of experts were asked to provide the topics they thought should be included to give adequate and complete information to adults with kidney transplantation.

To select the group of experts, the lead investigator contacted the nurses from 5 different specific kidney transplantation units that are currently operative in the public hospital system of the Community of Madrid, Spain.

During this first contact, nurses were asked to give their consent to receive information on the project, giving them the possibility to participate in such a project.

After obtaining their permission, they were sent an email including an invitation explaining how to participate in the project, information on its objectives, different phases, what their contribution would be, and the informed consent form to participate in it. To select the items that should be included in the guideline, a web-based open first round of questions was sent through email to all participants with all the items they thought should be included in the guideline.

Afterwards, a web-based survey was created that was later developed by the research team from January to April 2022, including two parts: (1) questions on the social and demographic data of the participants; and (2) questions on the content of the guideline; these questions were answered in step 3 of the process, once the bibliographical review that was the basis of the guide had been made.

### Second Step: Bibliographic Search

A review of the available medical literature was conducted to define the items that should be included in the information guideline for adults with kidney transplants. Through this review, the most relevant elements that should be taken into consideration regarding proper self-care practices in adults with kidney transplants were identified. Also, the most influential elements affecting the survival of the organ were identified. In addition, the compliance of patients with healthy habits was also assessed, as were the elements that might affect it.

The search was conducted in 3 different electronic databases: Medline/PubMed, Web of Science, and CUIDEN, from January 1, 2012, to December 31, 2022.

Search algorithms were based on the Population, Intervention, Comparator, Outcomes (PICO) strategy (population: adults with kidney transplants; intervention: any intervention aimed at promoting compliance with healthy lifestyles; comparator: no intervention or a different intervention; and outcomes: change in compliance with healthy lifestyles) [19] by selecting keywords using medical subject headings (MeSH) terminology [20]. The key terms used were “kidney transplant* / renal transplant*” and “lifestyle” plus “intervention” and “compliance.” Search strategies were adapted to databases by using different Boolean operators. Search was restricted to reports and manuscripts published over the past 10 years in such a way that all relevant studies that were not obsolete could be retrieved. The eligibility criteria were primary research studies written in any language and describing nonpharmacological interventions to improve compliance in adults with kidney transplants, and the lifestyles proposed by the medical literature. Only clinical trials were considered since only the effects of interventions were sought. No restrictions were put on the study duration or size of the sample. Reference lists of the included publications were also screened to identify additional articles. Reading and data mining were both conducted by 2 independent authors. Dissenting opinions were resolved through consensus.

Afterwards, based on the information provided by the group of experts and the information found in the bibliographic search, the information guideline for adults with kidney transplantation draft version 1 was created. Then, it was emailed to the panel of experts with a web-based survey.

### Third Step: Delphi Process With On-Site Phase

In the third step, a consensus group was created using the Delphi technique, followed by an on-site phase. The Delphi technique was based on the following 4 fundamental pillars [21]:

- An iterative process in which experts gave their opinion more than once was created. This gave them the opportunity to reflect on the opinions given based on the proposals made by others.
- Controlled feedback was given after each round, which allowed the free circulation of information inside the group, thus establishing a common language.
- Anonymity was kept during the first round and across the web-based survey, in the sense that none of the group members knew who had given a particular answer. However, and this is why the Delphi modified technique was used [22], this was not possible during the on-site discussion that, nonetheless, brought agility to the consensus process. We consider that this option, the face-to-face discussion, which represents a modification of the Delphi technique, has contributed to fostering the discussion and refining the subtleties of the responses. Textbox 1 depicts the characteristics involved in this modified Delphi technique.
- Finally, a globally calculated answer of the group was given, in which the individual results were included.

**Characteristics of the modified Delphi technique. Modified from Humphrey-Murto et al [22].**

1. Email questionnaires
2. Collect decisions anonymously
3. Obtain formal feedback as group ratings
4. Carry out face-to-face interactions
5. Carry out structured interactions

Therefore, to proceed with the modified Delphi technique, the items of the questionnaire—which was web-based—were elaborated to later move on to an on-site discussion.

The following questions were asked across the survey to assess each section of the guideline:

- Do you think that the topics of discussion in this section are appropriate? With a dichotomous response of yes or no, if the answer is negative, a box opens immediately with the following question: What would you add or suppress?
- Do you think that the message transmitted with the content of this section is appropriate? With a dichotomous response of yes or no, if the answer is negative, a box opens immediately with the following question: What would you change?
- Do you think it is long enough? With three response options: (1) yes; (2) no, it is too short; and (3) no, it is too long. If the answer is negative, a box opens immediately with the following question: What would you add or suppress?
- Do you think that the information provided is appropriate? With three different response options: (1) yes; (2) no, I think some information is missing; and (3) no, I think there is too much information. If the answer is negative, a box opens immediately with the following question: What would you change in the information provided?
- Do you think that the information provided is up-to-date? With a dichotomous response of yes or no, if the answer is negative, a box opens immediately with the following question: What part would you say is outdated? Please, if possible, provide current references.
- Rank from 0 to 4 the relevance of the information provided, with 0 being irrelevant and 4 being positively relevant.

The survey included a total of 105 questions. Given the limited number of participant experts, consensus was only achieved on items on which 4 of 5 respondents (80%) agreed, with mean scores >3.5 out of 4 possible points regarding the relevance of the information provided in each section.

In a third communication with respondents, the information guideline on adults with kidney transplantation draft version 1 was submitted, including a link to a web-based survey.

Finally, after the review that followed the web-based survey, the information guideline draft version 2 was created, followed by an on-site meeting with the group of experts.

### On-Site Discussion

Participants were summoned to an on-site meeting that was held at Hospital Clínico San Carlos de Madrid, Madrid, Spain, in October 2022. A total of 5 experts attended the meeting.

A semistructured interview was held at that meeting, where the lead investigator acted as the moderator to discuss topics on which no agreement had been reached. At the end, some time was given to express all comments, suggestions, and doubts deemed necessary by the experts.

After all these steps and their corresponding changes to the information provided, the guideline was edited by inserting images to make it more pleasing and enjoyable for the reader.

The final draft of the guideline was submitted, seeking the approval of the Madrid Health Authority Technical Coordination Commission. A positive report on the guideline was given by this commission.

### Ethical Considerations

Study participation was voluntary, and participants were given the option to withdraw their consent and walk away from the study at any time without any repercussions.

The project was conducted in full compliance with the latest iteration of the Declaration of Helsinki and the International Conference on Harmonization Good Clinical Practice Guideline (ICH GCP).

All personal data were tagged with a single code. Only the investigator was authorized to associate data with each participant. The lead investigator is responsible for guarding this information in a confidential way as long as legally required and in full compliance with the Spanish legislation: Organic Law 3/2018, of December 5, Protection of Personal Data and Guarantee of Digital Rights (LOPDGDD), and European legislation regarding data protection: Regulation (EU) 2016/679 of the European Parliament and of the Council of 27 April 2016 on the protection of natural persons with regard to the processing of personal data and on the free movement of such data, and repealing Directive 95/46/EC (General Data Protection Regulation).

This project was submitted to the Hospital Clínico San Carlos Research Ethics Committee as part of the clinical trial project to assess the impact of a nursing intervention on compliance with medication and healthy habits in adult patients with kidney transplantation (22/325-EC_X).

## Results

The mean age of the 5 participant nurses was 52.2 (SD 9.7) years, and 80% (4/5) were women with a median of time working as a nurse, at the nephrology unit, and at the kidney transplantation unit of 27 (IQR 19.5-30) years, 21 (IQR 7-24) years, and 3 (IQR 2.5-7.5) years, respectively.

Regarding training, 60% (3/5) were graduates or undergraduates, while 20% (2/5) were holders of a master’s degree in nephrology. They all had previously received specific training on kidney transplantation through continuing medical education courses.

In the first round, when an email was sent with an open question to elicit topics of interest that should be included in the information guideline on adults with kidney transplantation, 3 of the 5 nurses involved sent the information they were giving adults with kidney transplantation from their offices, while the remaining 2 responded to the open question with topics of discussion interesting to them and consistent with the information provided by the rest of the group.

After grouping the information provided by the group of experts and the information found in the medical literature available, the information guideline on adults with kidney transplantation draft version 1 was created, including 10 different sections or topics of discussion (Textbox 2).

Through the web-based survey, consensus data shown in Table 1 with all the questions from each section were obtained.

**Information guideline for adults with kidney transplantation draft version 1.**

**Table of contents**

1. Introduction: What does a kidney transplantation actually mean?
2. Drug therapy. Immunosuppressant drugs.
3. Recommendations and care
  Hygiene and skin care
  Food and diet
  Active way of life and exercise
  Transplant-related safety
    - Home
    - Pets
    - Gardening
4. Emotional status
5. Sexuality
6. Travels
7. Frequently asked question
8. In conclusion: recommendation and overall pieces of advice
9. Emergency. Reason for going to the hospital.
10. References

**Table 1:** Consensus measures reached after completing the web-based survey for each of the sections included in the information guideline for adults with kidney transplants (N=5).

Section	Distribution of scores for relevance of the information^a^						Consensus reached, %	Draft version 2 was changed
	0, %	1, %	2, %	3, %	4, %	Mean (SD)		
Title	N/A^b^	N/A	N/A	N/A	N/A	N/A	100	No
Introduction	0	0	0	20	80	3.8 (0.4)	80	No
Drug therapy	0	0	0	20	80	3.8 (0.4)	60	Yes, with sections pending the on-site meeting
Recommendations and care: hygiene and skin care	20	0	0	0	80	3.4 (1.34)	80	Yes, with sections pending the on-site meeting
Recommendations and care: Food and diet	0	0	20	0	80	3.6 (0.9)	60	Yes
Recommendations and care: Active way of life and exercise	0	0	20	20	60	3.4 (0.9)	80	Yes, with sections pending the on-site meeting
Recommendations and care: Transplant-related safety	0	0	20	0	80	3.6 (0.9)	80	No
Emotional status	0	0	0	20	80	3.8 (0.4)	100	No
Sexuality	0	20	0	20	60	3.2 (1.3)	80	Yes
Travels	0	0	20	0	80	3.6 (0.9)	80	No
Frequently asked questions	0	0	20	0	80	3.6 (0.9)	80	No
In conclusion	0	0	20	20	60	3.4 (0.9)	80	No
Emergency	0	0	20	20	60	3.4 (0.9)	60	Yes, with sections pending the on-site meeting
References	N/A	N/A	N/A	N/A	N/A	N/A	60	Yes, with sections pending the on-site meeting

^a^Assessed using a Likert scale, with 0=not relevant at all and 4=completely relevant.

^b^N/A: not applicable.

After reviewing the feedback received from the participants through the web-based survey, the contents of the sections on which consensus was reached regarding modifications were eventually updated. The format of the table of contents proposed in draft version 1 was kept and gave way to the information guideline for adults with kidney transplantation draft version 2. Sections on which experts did not agree with the information provided and no consensus was reached on what should be changed were proposed as topics of discussion during the on-site meeting and left for future change (Table 1).

Draft version 2 was discussed in the on-site meeting. After this meeting, the contents were updated once again. Draft version 3 is still pending. However, it will include the index that has not been modified and that coincides with Textbox 1 and all the content approved by consensus from the panel of experts.

After all these steps, it was decided to edit the guideline. Also, to achieve a more visually appealing guideline for the reader, original images created for this purpose were added by the author, who provided them for free.

## Discussion

### Overview

Health training and education are 2 of the nursing competencies most present in the entire health care process, as are prevention and the promotion of self-care at the base of these processes [23].

The beginning and development of this project took place for 2 main reasons. On the one hand, it was a response to the doubts and questions posed by health care personnel regarding compliance with healthy habits in adult patients with kidney transplants. On the other hand, it was born as a response to a growing demand for information from users who, thanks to patient-centered care and individual empowerment to improve one’s own health, have become more self-aware of the entire health-disease process.

As a reflection, this methodology for the creation of an effective guideline—agreed and based on the best evidence available—has been agile, useful, and allowed periodic updates with limited effort.

The usefulness of this guide will be evaluated in the clinical trial (ClinicalTrial.gov; NCT05715580), whose goal is to analyze the impact of an informative nursing intervention on adherence to drug treatment and a healthy lifestyle in patients with kidney transplants and the effect of poor adherence on clinical predictors of poor kidney function.

It seems evident that, beyond the imprint left by each health professional in his or her respective office or unit, having common tools and resources available promotes the development of similar health care strategies within a standardized system of health care provision.

### Limitations

One of the limitations in this study was the small group of experts that, although it included all nurses from the kidney transplantation units of the public network of the Community of Madrid, did not take into consideration other professional profiles. Also, given the method used to collect the experts, it was impossible to maintain the anonymity of the group.

On the other hand, a small group was good for obtaining the involvement and cooperation of participants during the entire process—something that was confirmed by the lack of withdrawals—which is typical of this kind of methodology.

Additionally, it is possible that the participants, as experts, only confirmed the relevance of the topics instead of considering all possible issues to address. This could potentially lead to confirmation bias, which could have been mitigated by involving patients or patient associations in the guide development process.

Finally, in the case of the responses requested from the group of experts in the survey, they may be biased by the “framing effect,” whereby the formulation, structure, and presentation of the questions could lead to changes in opinion or bias the response. This is a factor that needs to be taken into consideration [24].

### Conclusions

Thanks to the methodology used in this research, we managed to describe the habits that, based on the medical literature available and the opinion of experts, have played a key role in the good functioning of transplanted kidneys, thus impacting the survival of both the organ and the recipient.

Therefore, in the information guideline for adults with kidney transplants created as a result of this project, self-care practices identified as necessary and relevant to guarantee the success of transplantation were included.

Although other informative documents already existed for adults with kidney transplantation, the creation of this guideline allowed us to structure the necessary health care, thus generating a single document with all the necessary information and recommendations for patients to implement self-care measures effectively.

Therefore, a useful, easy-to-understand tool for health professionals in the field of kidney transplantation was created using simple language and with the latest scientific evidence available to date.

We hope this new tool will make the work of health professionals involved in kidney transplantations easier. At the same time, we hope it improves the quality of life of adult patients with kidney transplants as well as the quality of care they perceive.

## Abbreviations

ICH GCP: International Conference on Harmonization Good Clinical Practice
LOPDGDD: Organic Law on Protection of Personal Data and Guarantee of Digital Rights
MeSH: medical subject heading
PICO: Population, Intervention, Comparator, Outcomes

## Data Availability

The data sets generated during and/or analyzed during this work are available from the corresponding author on reasonable request.

## References

[1] Meier-Kriesche HU, Ojo AO, Port FK, Arndorfer JA, Cibrik DM, Kaplan B (2001). Survival improvement among patients with end-stage renal disease: trends over time for transplant recipients and wait-listed patients. J Am Soc Nephrol.

[2] Thiruchelvam PTR, Willicombe M, Hakim N, Taube D, Papalois V (2011). Renal transplantation. BMJ.

[3] Senanayake S, Graves N, Healy H, Baboolal K, Barnett A, Sypek MP, Kularatna S (2020). Donor kidney quality and transplant outcome: an economic evaluation of contemporary practice. Value Health.

[4] (2022). Actividad de donación y trasplante renal. Organizacion Nacional de Trasplantes.

[5] Pascazio L, Nardone IB, Clarici A, Enzmann G, Grignetti M, Panzetta GO, Vecchiet C (2010). Anxiety, depression and emotional profile in renal transplant recipients and healthy subjects: a comparative study. Transplant Proc.

[6] Oniscu GC, Brown H, Forsythe JLR (2004). How great is the survival advantage of transplantation over dialysis in elderly patients?. Nephrol Dial Transplant.

[7] Kenawy AS, Gheith O, Al-Otaibi T, Othman N, Atya HA, Al-Otaibi M, Nagy MS (2019). Medication compliance and lifestyle adherence in renal transplant recipients in Kuwait. Patient Prefer Adherence.

[8] (2022). Nutrition and transplant. National Kidney Foundation.

[9] Takahashi A, Hu SL, Bostom A (2018). Physical activity in kidney transplant recipients: a review. Am J Kidney Dis.

[10] Ronco C, Mason G, Karopadi AN, Milburn A, Hegbrant J (2014). Healthcare systems and chronic kidney disease: putting the patient in control. Nephrol Dial Transplant.

[11] Lopez V, Hernandez D, Gonzales M (2021). Resultados globales del trasplante renal. Nefrología al día.

[12] Calella P, Hernández-Sánchez S, Garofalo C, Ruiz JR, Carrero JJ, Bellizzi V (2019). Exercise training in kidney transplant recipients: a systematic review. J Nephrol.

[13] Estrategia de promoción de la salud y prevención en el SNS. Ministerio de Sanidad.

[14] Li Y, Pan A, Wang DD, Liu X, Dhana K, Franco OH, Kaptoge S, Di Angelantonio E, Stampfer M, Willett WC, Hu FB (2018). Impact of healthy lifestyle factors on life expectancies in the US population. Circulation.

[15] Lin SY, Fetzer SJ, Lee PC, Chen CH (2011). Predicting adherence to health care recommendations using health promotion behaviours in kidney transplant recipients within 1-5 years post-transplant. J Clin Nurs.

[16] Totti V, Campione T, Mosconi G, Tamè M, Tonioli M, Gregorini M, Scarpioni R, Storari A, Mignani R, Sella G, Bellis L, Cardillo M, Sangiorgi G (2020). Observational retrospective study on patient lifestyle in the pretransplantation and post-transplantation period in the Emilia-Romagna region. Transplant Proc.

[17] Totti V, Campione T, Mosconi G, Tamè M, Todeschini P, Sella G, Roi GS, Spazzoli A, Angelini ML, Sangiorgi G, Giannini A, Bellis L, Nanni Costa A (2019). Promotion of pre- and post-transplant physical exercise in the Emilia-Romagna region: the network of the program "transplantation, physical activity, and sport". Transplant Proc.

[18] Nowicka M, Górska M, Nowicka Z, Edyko K, Goździk M, Kurnatowska I (2021). Adherence to pharmacotherapy and lifestyle recommendations among hemodialyzed patients and kidney transplant recipients. J Ren Nutr.

[19] Schardt C, Adams MB, Owens T, Keitz S, Fontelo P (2007). Utilization of the PICO framework to improve searching PubMed for clinical questions. BMC Med Inform Decis Mak.

[20] MeSH (Medical Subject Headings). National Library of Medicine.

[21] Varela-Ruiza M, Díaz-Bravoa L, García-Durána G (2012). Descripción y usos del método Delphi en investigaciones del área de la salud. Investig en Educ Médica.

[22] Humphrey-Murto S, Varpio L, Wood TJ, Gonsalves C, Ufholz LA, Mascioli K, Wang C, Foth T (2017). The use of the Delphi and other consensus group methods in medical education research: a review. Acad Med.

[23] Lindberg M, Lundström-Landegren K, Johansson P, Lidén S, Holm U (2012). Competencies for practice in renal care: a national Delphi study. J Ren Care.

[24] Chong D, Druckman JN (2007). Framing theory. Annu Rev Polit Sci.

